# Ruthenium-Loaded Halloysite Nanotubes as Mesocatalysts for Fischer–Tropsch Synthesis

**DOI:** 10.3390/molecules25081764

**Published:** 2020-04-11

**Authors:** Anna Stavitskaya, Kristina Mazurova, Mikhail Kotelev, Oleg Eliseev, Pavel Gushchin, Aleksandr Glotov, Ruslan Kazantsev, Vladimir Vinokurov, Yuri Lvov

**Affiliations:** 1 Gubkin University, 65 Leninsky Prosp., Moscow 119991, Russia; mazurovachris55@mail.ru (K.M.); kain@inbox.ru (M.K.); oleg@ioc.ac.ru (O.E.); guschin.p@mail.ru (P.G.); glotov.a@gubkin.ru (A.G.); vinok_ac@mail.ru (V.V.); 2 N.D. Zelinsky Institute of Organic Chemistry, 47 Leninsky Prosp, Moscow 119991, Russia; rvk7@mail.ru; 3 Institute for Micromanufacturing, Louisiana Tech University, 505 Tech Drive, Ruston, LA 71272, USA

**Keywords:** halloysite, nanotube, ruthenium, nanoparticle, Fischer–Tropsch, hydrocarbons, alkanes, catalysis

## Abstract

Halloysite aluminosilicate nanotubes loaded with ruthenium particles were used as reactors for Fischer–Tropsch synthesis. To load ruthenium inside clay, selective modification of the external surface with ethylenediaminetetraacetic acid, urea, or acetone azine was performed. Reduction of materials in a flow of hydrogen at 400 °C resulted in catalysts loaded with 2 wt.% of 3.5 nm Ru particles, densely packed inside the tubes. Catalysts were characterized by N_2_-adsorption, temperature-programmed desorption of ammonia, transmission electron microscopy, X-ray fluorescence, and X-ray diffraction analysis. We concluded that the total acidity and specific morphology of reactors were the major factors influencing activity and selectivity toward CH_4_, C_2–4_, and C_5+_ hydrocarbons in the Fischer–Tropsch process. Use of ethylenediaminetetraacetic acid for ruthenium binding gave a methanation catalyst with ca. 50% selectivity to methane and C_2–4_. Urea-modified halloysite resulted in the Ru-nanoreactors with high selectivity to valuable C_5+_ hydrocarbons containing few olefins and a high number of heavy fractions (α = 0.87). Modification with acetone azine gave the slightly higher CO conversion rate close to 19% and highest selectivity in C_5+_ products. Using a halloysite tube with a 10–20-nm lumen decreased the diffusion limitation and helped to produce high-molecular-weight hydrocarbons. The extremely small C_2_–C_4_ fraction obtained from the urea- and azine-modified sample was not reachable for non-templated Ru-nanoparticles. Dense packing of Ru nanoparticles increased the contact time of olefins and their reabsorption, producing higher amounts of C_5+_ hydrocarbons. Loading of Ru inside the nanoclay increased the particle stability and prevented their aggregation under reaction conditions.

## 1. Introduction

Global consumption of transportation fuels, lubricants, and chemicals amid the depletion of petroleum reserves is leading to a growing demand for alternative hydrocarbon sources. These non-petroleum feedstocks include natural gas and coal, as well as renewable biomass and urban wastes [1]. All of them can be converted into clean hydrocarbons via syngas production followed by Fischer–Tropsch synthesis (FTS).

Iron- and cobalt-based microcatalysts are currently used in commercial Fischer–Tropsch (FT) processes mainly due to their low cost and relatively high efficiency. However, ruthenium is known to be the most active catalyst which can work without promoters, and it typically produces heavier hydrocarbons [2,3]. Activity of ruthenium in CO hydrogenation depends on the particle size with 5–10-nm nanoparticles being the most active [4,5].

In recent years, nanostructured systems including those based on metal ruthenium were actively studied for their higher reaction rate and selectivity for the main products [6,7,8,9,10]. Commonly used supports for FT catalysts are silica, alumina, zirconia, and titanium oxide [5,11,12]. Alumina-supported catalysts could give higher ruthenium dispersion compared to a silica carrier, while SiO_2_-based catalysts are characterized by low acidity resulting in higher long-chain hydrocarbon selectivity [13]. For carbon nanotubes as a carrier in the Fischer–Tropsch synthesis, it was shown that placing metal particles inside the nanotubes leads to an increase in the efficiency of the catalysis [12,13,14,15]. 

Halloysite is a unique mineral that is formed by rolling of kaolin sheets into multiwalled mesoporous nanotubes [16]. The halloysite lumen’s surface is formed by alumina, and the outer tube’s surface contains silica. Such a structure allows for site-dependent chemistry exploiting a negatively charged outer surface and positive inner lumen. Low acidity is another property of halloysite that is important for FTS catalysts [5]. Recently, we showed that metal nanoparticles could be synthesized selectively inside halloysite using azines as complexing agents [17]. Ru loaded inside halloysite by complexation of RuCl_3_ with 1,2-bis(2-furylmethylene)hydrazine was found to be efficient in the hydrogenation of mono-aromatics [18,19,20]. No works were performed on the complexing agent’s effect on morphology, physico-chemical properties, and catalytic activity of ruthenium-loaded halloysite. 

For the first time, we propose natural mesoporous halloysite clay nanotubes as a carrier for ruthenium-containing catalysts for producing hydrocarbons from synthesis gas (CO + H_2_). There was a previous study on halloysite-supported cobalt Fischer–Tropsch synthesis catalysts [21]. We showed that Ru/halloysite core–shell nanocatalysts for CO hydrogenation allowed for much higher catalytic efficiency. We also demonstrated how the choice of complexing agents affects activity of halloysite-based catalysts in Fischer–Tropsch synthesis.

## 2. Results and Discussion

### 2.1. Structure, Morphology, and Composition of Ru-Loaded Halloysite Catalysts 

Halloysite is based on rolled kaolin plates forming tubes of 50–60 nm outer diameter with a 12–15-nm-diameter inner lumen and a length of 500–800 nm. These nanotubes have different inside/outside chemistry composed of Al_2_O_3_ and SiO_2_, exhibiting different chemical reactivities. This allowed for selective tube lumen loading with metal ions [16]. 

Halloysite nanotubes with ruthenium nanoparticles inside were obtained using prior tubes loaded with various organic ligands with a further ruthenium salt solution socking inside the tube due to favorable metal–ligand complex formation and its reduction. To prevent nanoparticle formation on the outer surface, washing of excess of reagents was performed after each synthesis procedure. Ethylenediaminetetraacetic acid (EDTA) was chosen for HNT@Ru-1 preparation, urea was chosen for HNT@Ru-2, and acetone azine was chosen to obtain HNT@Ru-3. Organic molecules were intercalated inside nanotubes as described in the experimental section. To achieve a ruthenium concentration of ca. 2%, a two-step ruthenium chloride loading technique was modified from our previous work [17]. Before Fischer–Tropsch experiments, materials were reduced in a flow of H_2_ for 3 h at 400 °C. 

**Figure 1:**
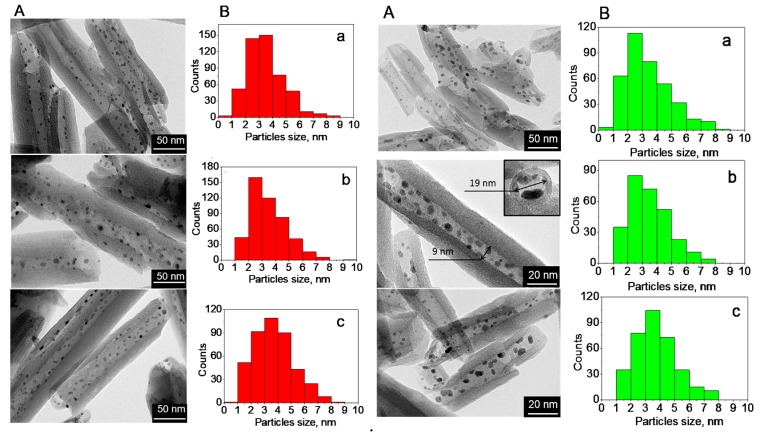
TEM image (**A**) and particle size distribution (**B**) of fresh pristine halloysite (HNT)@Ru-1 (a), HNT@Ru-2 (b), and HNT@Ru-3 (c) (left), and spent HNT@Ru-1 (a), HNT@Ru-2 (b), and HNT@Ru-3 (c) (right).

The morphology of catalysts before and after Fischer–Tropsch reaction is shown in Figure 1 together with particle size distribution calculated from transmission electron microscopy (TEM) images. Selective ruthenium loading could be seen in all samples with a homogeneous nanoparticle distribution inside the aluminosilicate nanotubes. Particle size was similar, in the range of 1–8 nm with maxima between 2 and 4 nm and a particle size of 3.5 nm (Table 1). TEM of catalysts after Fischer–Tropsch synthesis showed no significant influence of reaction conditions on ruthenium particle distribution inside the nanotubes, as well as on their size. The stability of nanoparticles was reached due to their loading inside the tubes.

Si, Al, and Ru concentrations in pristine halloysite (HNT) and catalysts are shown in Table 1. It is known that the internal surface of halloysite is composed of alumina, while the external surface is composed of silica, whereby the concentration of Si and Al may be changed with acid or alkaline treatment [22]. To evaluate the chemical composition changes inside the nanotube, we used the Si/Al ratio calculated from elemental analysis data and observed changes; we obtained values of 0.93, 0.99, 0.93, and 0.96, respectively, for HNT, HNT@Ru-1, HNT@Ru-2, and HNT@Ru-3. We assumed that a small amount of Al was etched from samples HNT@Ru-1 and HNT@Ru-3. Ruthenium concentration was determined to be 2.0 wt.% for HNT@Ru-1 and HNT@Ru-2, and 2.2 wt.% for HNT@Ru-3 (Table 1).

**Figure 2:**
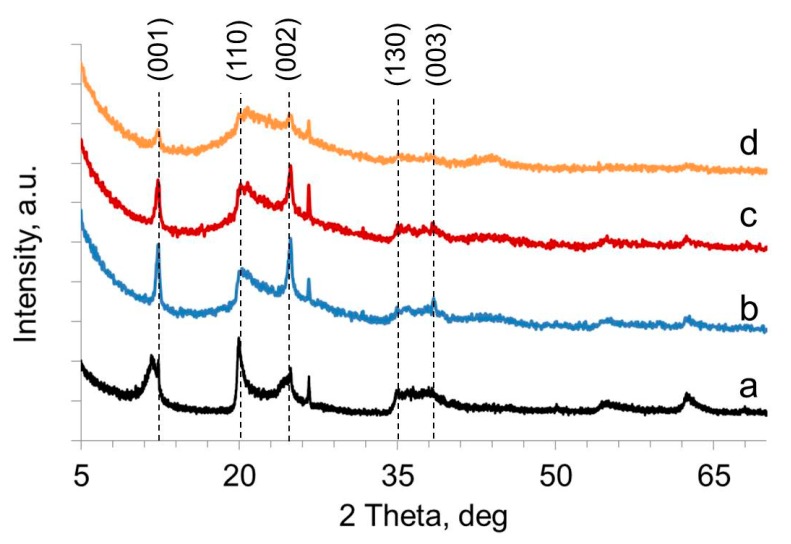
X-ray diffraction (XRD) patterns for the HNT (**a**), HNT@Ru-1 (**b**), HNT@Ru-2 (**c**), and HNT@Ru-3 (**d**).

X-ray diffractograms of pristine halloysite and halloysite nanotubes selectively loaded with ca. 2 wt.% of Ru are shown in Figure 2. The peak at 2θ = 12.36° is characteristic of dehydrated halloysite (d_001_ = 0.7 nm) [23,24]. The intensity of 12.36° (d_001_), 20.12° (d_110_), and 25.28° (d_002_) peaks decreased in the order HNT > HNT@Ru-1 ≥ HNT@Ru-2 > HNT@Ru-3. The halloysite-7Å was preserved in HNT@Ru-1 modified with ethylenediaminetetraacetic acid and urea-modified HNT@Ru-2. In the case of HNT@Ru-3 modified with acetone azine, the broadening of 12.35°, 20.12°, and 25.28° reflections belonging to the (001), (100), and (002) facets of halloysite revealed the amorphization of halloysite and formation of metahalloysite [25]. Generally, halloysite amorphization occurs during the dehydroxylation stage at 450 °C. In the case of modified halloysite, dehydroxylation partly occurred at 400 °C [26,27,28]. Ethylenediaminetetraacetic acid is too large to intercalate, and urea does not have a significant influence on dehydroxylation temperature. There are no prior studies on the influence of intercalated acetone azine on dehydroxylation temperature; we propose that it activated the interlayer hydroxyls and led to a decrease in the transition temperature. From Figure 1, it can be seen that, after thermal treatment and reaction, the clay tubular morphology was preserved. Due to the low concentration and small crystalline size of Ru nanoparticles (less than 5 nm), as well as the overlapping of signals, their presence was not confirmed by X-ray diffraction (XRD).

**Figure 3:**
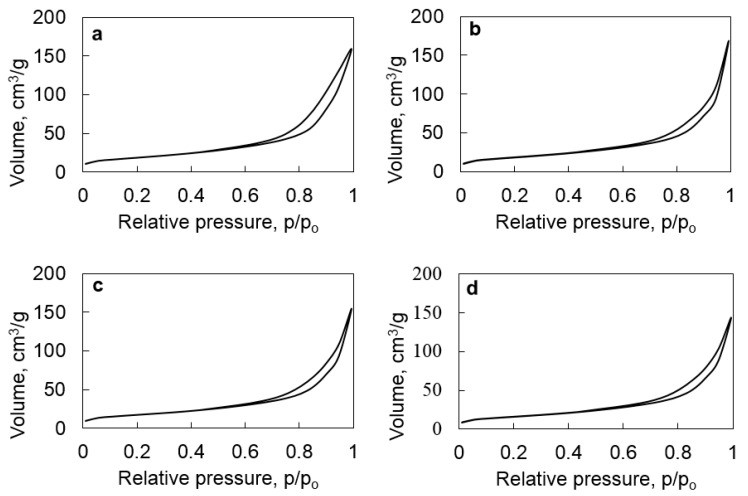
N_2_ adsorption–desorption isotherms of HNT (**a**), HNT@Ru-1 (**b**), HNT@Ru-2 (**c**), and HNT@Ru-3 (**d**).

The specific surface areas of halloysite and HNT@Ru-1, HNT@Ru-2, and HNT@Ru-3 were determined using low-temperature nitrogen adsorption/desorption. The data obtained are summarized in Table 1 and varied in the range of 58 to 66 m^2^/g. Figure 3 shows that the samples had typical type IV isotherms of mesoporous materials [29,30]. The porous structure of halloysite was preserved after modification and deposition of ruthenium particles inside the lumen.

**Figure 4:**
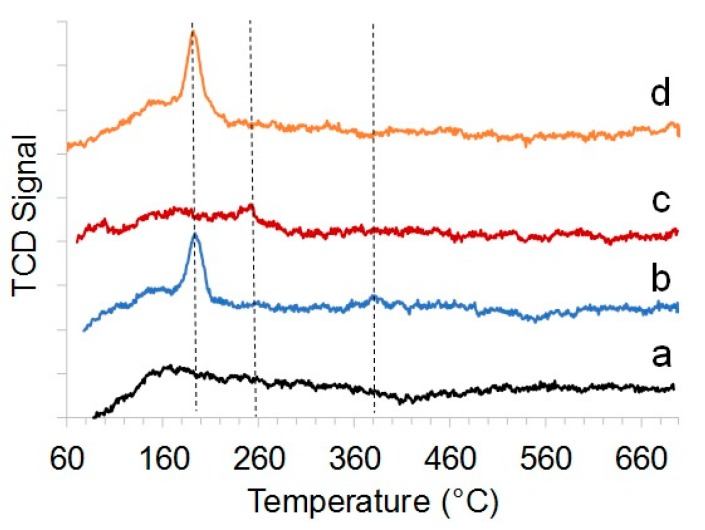
NH_3_ temperature-programmed desorption (TPD) profiles of the HNT (**a**), HNT@Ru-1 (**b**), HNT@Ru-2 (**c**), and HNT@Ru-3 (**d**).

Analysis of the temperature-programmed desorption of ammonia (TPD) profiles was conducted. The thermal desorption curves of HNT@Ru-1 and HNT@Ru-3 had a major desorption area at 170–200 °C corresponding to weak acid sites (Figure 4). From TPD-NH_3_ curves, it can be seen that HNT@Ru-1 had an intensive peak at 190 °C and a tiny peak at 350 °C. HNT@Ru-2 had a very small peak at 260 °C corresponding to moderate acid sites. HNT@Ru-3 TPD-NH_3_ also showed the presence of mainly weak acid centers. The total number of acid sites in comparison with the pristine halloysite increased in the samples HNT@Ru-1 by 137 µmol/g NH_3_ and HNT@Ru-3 by 72 µmol/g NH_3_, whereas, for HNT @Ru-2, it decreased by 49 units (Table 1). Based on the obtained data (Table 1), we can conclude that the acidity decreased in the order HNT@Ru-1 > HNT@Ru-3 > HNT > HNT@Ru-2.

### 2.2. Catalytic Efficiency of Ru-Loaded Halloysite Catalysts in Fischer–Tropsch Synthesis 

The Fischer–Tropsch synthesis was carried out in a fixed-bed flow-type reactor at 260 °C. After 32 h on stream, pseudo steady-state conditions were reached under which the concentrations of all products were determined. Catalyst performance was evaluated in terms of CO conversion and selectivity to particular products. Furthermore, ruthenium–time yield was calculated as amount of CO converted over one mole of Ru per second.

Carbon monoxide conversion slightly increased in the order HNT@Ru-1 < HNT@Ru-2 < HNT@Ru-3, although ruthenium–time yield values were very close for all three samples (Table 2). The conversion of CO on HNT@Ru-3 was slightly higher than for the other two catalysts. Considering that the actual Ru content in this catalyst was 10% higher than in the rest, the specific activity of ruthenium (Ru–time yield) was not the highest. Apparently, amorphization of the carrier in this catalyst does not affect activity. The synthesis products were only hydrocarbons, and the yield of oxygen-containing compounds was negligible. Notably, CO conversion into CO_2_ was at the limit of gas chromatography determination, indicating the very low activity of the catalysts in the water gas shift reaction.

Depending on the ligand being used for the catalyst preparation, CH_4_ selectivity varied widely from 19.9% to 52.8%. HNT@Ru-1 behaved as a methanation catalyst rather than FTS catalyst, accompanied by the highest total acidity values (Table 1). The highest selectivity to the most valuable C_5+_ hydrocarbons [30] was obtained over HNT@Ru-2, with the lowest methane and light-hydrocarbon selectivity. Yields of methane and light hydrocarbons obviously correlated with catalyst total acidity (Table 1). We attribute this to the acid-catalyzed secondary reactions of synthesized higher hydrocarbons, such as cracking and hydrogenolysis [31,32].

**Figure 5:**
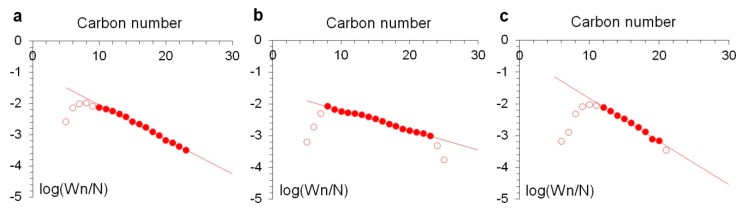
Anderson–Schulz–Flory (ASF) plots of hydrocarbon formation over HNT@Ru-1 (**a**), HNT@Ru-2 (**b**), and HNT@Ru-3 (**c**).

The molecular weight distribution of synthesized hydrocarbons obeyed the Anderson–Schulz–Flory (ASF) formula [33] (Figure 5). Some discrepancies with the ASF distribution in the field of low carbon numbers may be partly considered as an experimental artefact arising from the entrainment of light hydrocarbons with gas flow from the liquid product collector. Another probable reason was the readsorption of light olefins and their insertion into growing hydrocarbon chains, as well as the initiation of new chains [31,32]. Olefins are widely considered as primary FTS products [34,35,36]. The extremely low selectivity of HNT@Ru-2 and HNT@Ru-3 to C_2_–C_4_ hydrocarbons and the absence of olefins were indirect confirmations of the olefin reactions. The lowest percentage of olefins in C_5+_ products was obtained with HNT@Ru-2, which provided the highest ASF α value. We posit that the dense packing of Ru particles inside the halloysite lumen led to the re-adsorption of olefins, with subsequent hydrogenation and chain growth.

Thus, Ru-loaded halloysite catalysts provided considerable activity in CO hydrogenation, while their selectivity depended greatly on the preparation method. Urea-modified halloysite resulted in catalysts with high selectivity to valuable C_5+_ hydrocarbons, containing few olefins and a high number of heavy fractions.

For all tested catalysts, stationary conditions were achieved with no changes in CO conversion after 32 h on stream. The absence of changes in the dispersion of ruthenium in spent catalyst samples compared to the fresh ones confirmed that the catalysts were stable (Figure 1).

## 3. Materials and Methods 

### 3.1. Materials

Aluminosilicate nanotubes (Al_2_Si_2_O_5_(OH)_4_) (Sigma-Aldrich, St. Louis, MO, USA), ethylenediaminetetraacetic acid (EDTA) (C_10_H_16_N_2_O_8_) (Sigma-Aldrich) ≥99.5%, urea ((NH_2_)_2_CO) (RusChem, Moscow, Russia), hydrazine hydrate solution (N_2_H_4_*H_2_O) 78%–82% (RusChem), ruthenium chloride (RuCl_3_) (Aurat, Moscow, Russia), and sodium borohydride powder (NaBH_4_) (RusChem) were used in this study.

### 3.2. Catalyst Preparation 

Halloysite (1 g) and ethylenediaminetetraacetic acid (EDTA) (0.5 g) were dispersed in deionized water (30 mL) under ultrasound for 1 h. The resulting mixture was centrifuged (5500 rpm for 3 min) and washed three times with water to remove excess EDTA. The modified halloysite and ruthenium chloride (20 mg) were dispersed in ethanol (30 mL) for 30 min, and the dispersion was centrifuged (5500 rpm for 3 min). The precipitate was separated and washed with alcohol. Then, an aqueous solution of NaBH_4_ was added to reduce Ru^3+^. After completion of the reaction and degassing, the mixture was centrifuged and washed with water three times to remove by-products. The resulting precipitate was redispersed with ruthenium chloride (20 mg) in an alcohol medium, followed by washing and reduction of ruthenium complexes with NaBH_4_ to obtain metal in an amount of 2% by weight of halloysite. In the last step, the sample was dried at 65 °C for 24 h. As a result, HNT@Ru-1 was obtained.

To obtain HNT@Ru-2, the same procedure was performed; however, instead of ethylenediaminetetraacetic acid loading inside the tube, urea was used as a ligand. Halloysite (1 g) was dispersed in an aqueous solution of urea (30 mg/mL) under ultrasound for 1 h. 

To obtain HNT@Ru-3, halloysite (1 g) was dispersed in hydrazine hydrate (20 mL) under ultrasound for 30 min. The resulting mixture was centrifuged (5500 rpm for 3 min), and the precipitate was separated and washed with ethanol three times to remove excess hydrazine hydrate. Then, acetone (20 mL) was gradually added to the precipitate and kept in an ultrasonic bath for 30 min. The reaction of acetone with hydrazine produced acetone azine. The reaction mixture was centrifuged and washed three times with acetone. Modified halloysite and ruthenium chloride (20 mg) were dispersed in acetone (30 mL) for 30 min to form ruthenium complexes inside aluminosilicate nanotubes. Then, the dispersion was centrifuged (5500 rpm for 3 min), and the precipitate was separated and washed with acetone. Next, an aqueous solution of NaBH_4_ was added to reduce Ru^3+^. After completion of the reaction and degassing, the mixture was centrifuged and washed with water three times to remove by-products. The resulting precipitate was redispersed with ruthenium chloride (20 mg) in acetone, followed by washing and reduction of ruthenium complexes with NaBH_4_. In the last step, the sample was dried at 35 °C for 24 h.

Prior to catalytic reaction, the catalysts were activated at normal pressure in a hydrogen flow at 400 °C for 4 h.

### 3.3. Catalyst Characterization

Textural characteristics of the samples obtained were determined on a Micromeritics Gemini VII 2390t instrument (Micromeritics Instrument Corp., Norcross, GA, USA) using the low-temperature N_2_-adsorption method. Before measurements, the samples were degassed at a temperature of 300 °C for 4 h. The specific surface area was calculated using the Brunauer–Emmett–Teller (BET) equation with adsorption data in the relative pressure range P/P_0_ = 0.05–0.35.

The acidity of the samples was evaluated using the temperature-programmed desorption of ammonia (NH_3_-TPD) technique on a Micromeritics AutoChem HP2950 instrument (Micromeritics Instrument Corp., Norcross, GA, USA). The sample weighed ~0.3 g; it was placed in a quartz reactor and kept in a nitrogen flow at 700 °C for 1 h with a rate of 50 mL·min^–1^. Saturation was carried out in a flow of dried ammonia diluted with nitrogen (8 vol.% NH_3_) at a temperature of 60 °C for 30 min. The removal of physically adsorbed ammonia was carried out at 100 °C in a nitrogen flow of 50 mL·min^–1^ for 30 min. To obtain the NH_3_-TPD curve, the temperature was gradually raised from 100 °C to 700 °C at a rate of 10 °C·min^–1^. Samples of fresh and spent catalysts were examined using a transmission electron microscope (JEM-2100, JEOL, Tokyo, Japan). The obtained images were processed using the Image J program developed by employees of the National Institutes of Health (Madison, WI, USA.) Particle size distribution was analyzed using 4–5 microphotographs based on counting 400–500 nanoparticles. The processed data were used to plot the histograms and determine the average particle size of the metal with Origin Software (8, Northampton, MA, USA). 

Elemental analysis was performed on an ARL Quant’X energy-dispersive spectrometer (Thermo Fisher Scientific, Waltham, MS, USA) in air. The results were processed using the standard-less UniQuant method.

X-ray structural analysis (XRD) of the samples was carried out on a X-Ray Diffractometer (Rigaku SmartLab, Tokyo, Japan) in the 2θ range of 5°–80° at a speed of 5° per minute. Qualitative and quantitative analysis of the obtained diffraction patterns was carried out using the Rigaku PDXL software (Rigaku SmartLab, Tokyo, Japan) using the ICDD (International Center for Diffraction Data) and AMCSD (American Mineralogist Crystal Structure Database) databases.

### 3.4. Catalytic Experiment

The catalytic activity was studied in a Fischer–Tropsch laboratory synthesis fixed-bed unit with a stainless-steel flow integral reactor with an internal diameter of 14 mm. The catalyst (0.4 g) was mixed with 1 cm^3^ of quartz sand to avoid local overheating during hydrocarbon synthesis, and this mixture was loaded into the isothermal zone of the reactor. The activated catalysts were tested in the synthesis of hydrocarbons at a ratio of CO/H_2_ = 1/2, pressure = 1 MPa, and gas flow rate = 10 nL/(h × g_cat_). Temperature was gradually increased from 210 to 260 °C. After approximately 32 h at 260 °C, pseudo steady-state conditions were reached and catalytic performance (activity, selectivity) was evaluated.

Gas products (C_1_–C_4_ hydrocarbons, CO_2_) were analyzed with an LKM-80 gas chromatograph (1 m × 3 mm columns filled with molecular sieves, with helium as a carrier gas and a thermal conductivity detector, (LKM, Moscow, Russia)). C_5+_ liquid hydrocarbons were collected in a container at ambient temperature and analyzed with a Biochrome-1 instrument (quartz capillary column 50 m × 0.25 mm, with nitrogen as a carrier gas and a flame ionization detector, (LKM, Moscow, Russia)). The selectivity of C_5+_ was calculated using the difference in the total balance of mass and the quantity of gases C_1_–C_4_ and CO_2_.

## 4. Conclusions

For the first time, halloysite nanotubes selectively loaded with Ru nanoparticles via metal–organic complex reduction inside the lumen were used as catalysts for Fischer–Tropsch synthesis. A methanation catalyst with increased acidity compared to pristine nanotubes was produced when ethylenediaminetetraacetic acid was used for Ru complex formation inside nanotubes. The Ru nanocatalyst formed through urea assistant loading showed the highest selectivity to the most valuable C_5+_ hydrocarbons with α = 0.87. The highest olefin content in the C_5+_ fraction was reached with halloysite modified with acetone azine prior to ruthenium loading. No morphology changes were observed after 32 h on steam, confirming the stability of catalysts produced.

## Figures and Tables

**Table 1:** Physico-chemical characteristics of Ru-loaded halloysite catalysts. BET—Brunauer–Emmett–Teller.

Catalyst	Elemental Composition, wt. %			Surface Area, BET, m^2^/g	Average Particles Size (TEM), nm	Total Acidity, µmol/g
	**Si**	**Al**	**Ru**			
HNT	23.6	25.3	-	66	None	178
HNT@Ru-1	23.5	23.8	2.0	63	3.5	315
HNT@Ru-2	23.0	24.0	2.0	60	3.5	129
HNT@Ru-3	22.5	24.3	2.2	58	3.5	250

**Table 2:** Ru-loaded halloysite catalyst performance in Fischer–Tropsch synthesis ^1^.

Parameter	HNT@Ru-1	HNT@Ru-2	HNT@Ru-3
**CO conversion, %**	15.6	17.8	18.8
**Ru–time yield × 10^3^, mol_CO_/(mol_Ru_ s)**	29.3	33.4	32.1
**CH_4_ selectivity, %**	52.8	19.9	28.8
**C_2_–C_4_ selectivity, %**	20.3	1.6	3.2
**C_5+_ selectivity, %**	26.7	78.0	67.7
**CO_2_ selectivity, %**	0.2	0.5	0.3
**% olefins in C_5+_**	19.2	12.9	26.5
**ASF α**	0.78	0.87	0.73

^1^ Reaction conditions: P = 1.0 MPa, T = 260 °C, H_2_/CO = 2:1, gas flow rate = 10 nL/(h g_cat_); data were collected after 32 h on steam. ASF—Anderson–Schulz–Flory.
